# Draft Genome Sequences of 80 Salmonella enterica Serovar Infantis Strains Isolated from Food, Environmental, Human, and Veterinary Sources in Brazil

**DOI:** 10.1128/MRA.00313-21

**Published:** 2021-06-17

**Authors:** Felipe Pinheiro Vilela, Bruno Rocha Pribul, Dália dos Prazeres Rodrigues, Maria Balkey, Marc Allard, Juliana Pfrimer Falcão

**Affiliations:** a Faculdade de Ciências Farmacêuticas de Ribeirão Preto—USP, Departamento de Análises Clínicas, Toxicológicas e Bromatológicas, Ribeirão Preto, São Paulo, Brazil; b Fundação Oswaldo Cruz—FIOCRUZ, Rio de Janeiro, Rio de Janeiro, Brazil; c Division of Microbiology, Office of Regulatory Science, Center for Food Safety and Applied Nutrition, U.S. Food and Drug Administration, College Park, Maryland, USA; University of Maryland School of Medicine

## Abstract

Salmonella enterica serovar Infantis is a broadly distributed serovar infecting humans and animal reservoirs globally. Here, we report 80 draft genome sequences of *S*. Infantis strains isolated from diverse sources in Brazil. These data will improve our understanding of the specific traits of *S.* Infantis isolated in this country.

## ANNOUNCEMENT

Infections caused by nontyphoid Salmonella enterica serovars are considered one of the four major causes of foodborne diseases worldwide, accounting for 93.8 million cases of gastroenteritis and 155,000 deaths each year (1). Salmonella enterica subsp. *enterica* serovar Infantis is a globally reported host-unspecific serovar, capable of infecting mainly food-producing animals such as poultry, swine, and bovines and responsible for causing gastroenteritis in humans through the consumption of contaminated water and raw or undercooked meat products (2–5).

In this report, we announce a total of 80 draft genome sequences of Salmonella Infantis strains isolated from food, human, environmental, and veterinary sources between 2013 and 2018 in Brazil.

The strains were acquired from the Salmonella collection of the National Reference Laboratory for Enteric Diseases at the Oswaldo Cruz Foundation (FIOCRUZ) of Rio de Janeiro, where they were stored in phosphate-buffered agar at room temperature and/or in brain heart infusion (BHI) broth/glycerol at –70°C until use. Genomic DNA was extracted from the strains as previously described (6). Briefly, the strains were reactivated from storage in BHI broth and incubated overnight at 37°C. A total of 4 ml of bacterial growth was pelleted and treated with solution 1 (20% sucrose; 50 mM Tris-HCl, pH 8.0; and 50 mM EDTA), followed by treatment with solution 2 (50 mM NaCl, 1% sarkosyl, and 0.005 mg/ml of proteinase K). DNA separation was performed with phenol, chloroform, and isoamyl alcohol. The concentration was determined using a UV light spectrophotometer at 260 and 280 nm.

Libraries were prepared using 1 ng of genomic DNA with the Nextera XT DNA library preparation kit (Illumina, San Diego, CA). The genomes were sequenced in an Illumina MiSeq sequencer using the 2 × 150-bp paired-end MiSeq reagent kit version 3 (Illumina) according to the manufacturer’s recommendations. Quality control was performed using the MicroRunQC workflow in the Galaxy platform (7). *De novo* assemblies were generated from all Illumina sequence data using the SKESA version 2.2 assembler (8). The contigs for each isolate (draft genome sequences) were annotated using NCBI’s Prokaryotic Genome Annotation Pipeline (PGAP) (9). Default parameters were used for all software, except where otherwise noted. The genome sizes ranged from 4.6 to 5.2 Mb, the number of contigs per assembly for each isolate ranged from 30 to 96, and the C+G content ranged from 51.1 to 52.4%.

The data obtained will improve our understanding of the specific traits of Salmonella Infantis strains isolated in Brazil from multiple sources between 2013 and 2018. They will also provide support for future research regarding the *S.* Infantis phylogenetics, epidemiology, virulence, and antimicrobial resistance gene content, which will be detailed in future publications.

### Data availability.

The draft genome sequences of the 80 Salmonella Infantis isolates reported here are available in GenBank under the accession numbers listed in Table 1.

**TABLE 1:** Metadata of the 80 sequenced Salmonella Infantis strains isolated from food, environmental, human, and veterinary sources between 2013 and 2018 in Brazil

Collection strain no.	State of isolation^*a*^	Isolation source^*b*^	Isolation material	CFSAN strain no.	GenBank accession no.	SRA accession no.	No. of contigs	Genome size (Mb)	*N*_50_ (bp)	G + C content (%)	Coverage (×)
SI 1348/13	PR	Human feces	Human	CFSAN107127	AAWRHH000000000.1	SRX9368315	30	4,685,354	397,894	52.3	72
SI 2385/13	PR	Soy	Food	CFSAN107129	AAWRGU000000000.1	SRX9368322	39	4,639,975	214,277	52.3	35
SI 2950/13	AL	Human feces	Human	CFSAN107130	AAWRHS000000000.1	SRX9368227	37	4,694,965	275,151	52.3	62
SI 2951/13	AL	Human feces	Human	CFSAN107131	AAWRHN000000000.1	SRX9368262	35	4,619,292	397,894	52.3	75
SI 3156/13	SC	Disposable shoe cover	Environment	CFSAN107132	AAWRGH000000000.1	SRX9368326	57	4,849,812	263,497	52	82
SI 5025/13	SC	Human feces	Human	CFSAN107133	AAWRGA000000000.1	SRX9368380	45	4,730,564	222,277	52.2	59
SI 124/14	RS	Swine feces	Animal	CFSAN107134	AAWRDW000000000.1	SRX9368796	67	4,796,946	309,796	51.1	43
SI 210/14	SC	Dragging swab	Environment	CFSAN107136	AAWREM000000000.1	SRX9368701	60	4,745,933	328,256	52.2	34
SI 212/14	SC	Dragging swab	Environment	CFSAN107137	AAWRDZ000000000.1	SRX9368791	66	4,887,562	333,338	52.2	88
SI 388/14	SP	Soybean animal meal	Animal rations	CFSAN107138	AAWRER000000000.1	SRX9368696	31	4,756,799	526,057	52.1	58
SI 583/14	SC	Chicken carcass	Food	CFSAN107139	AAWREP000000000.1	SRX9368699	53	4,785,260	393,086	52.3	66
SI 584/14	SC	Pasta containing ham	Food	CFSAN107140	AAWREX000000000.1	SRX9368685	51	4,761,334	333,340	52.3	50
SI 677/14	SC	Carcass cleaning wipe	Food	CFSAN107141	AAWRFG000000000.1	SRX9368438	53	4,785,741	249,185	52.2	35
SI 723/14	SC	Dragging swab	Environment	CFSAN107142	AAWRFD000000000.1	SRX9368439	47	4,739,027	332,890	52.2	60
SI 982/14	RS	Chicken feces	Animal	CFSAN107143	AAWRHV000000000.1	SRX9368215	67	4,843,644	249,768	52.2	55
SI 1143/14	RS	Chicken feces	Animal	CFSAN107144	AAWRHU000000000.1	SRX9368225	45	4,935,012	376,680	52.3	108
SI 1284/14	SC	Dragging swab	Environment	CFSAN107145	AAWRIM000000000.1	SRX9366807	60	4,839,954	333,338	52.2	68
SI 1380/14	RS	Chicken feces	Animal	CFSAN107146	AAWRIF000000000.1	SRX9366813	48	4,747,908	276,149	52.3	52
SI 1408/14	RS	Human feces	Human	CFSAN107148	AAWRIL000000000.1	SRX9366809	31	4,639,197	332,890	52.4	66
SI 1409/14	RS	Human feces	Human	CFSAN107149	AAWRHF000000000.1	SRX9368319	34	4,624,103	263,497	52.2	52
SI 1441/14	RS	Mayonnaise	Food	CFSAN107150	AAWRHL000000000.1	SRX9368265	32	4,696,002	275,485	52.3	66
SI 1711/14	RS	Chicken feces	Animal	CFSAN107151	AAYKFJ000000000.1	SRX9741195	96	5,114,771	193,312	52.2	119
SI 2378/14	SC	Truck swab	Environment	CFSAN107152	AAWRHR000000000.1	SRX9368259	70	4,879,261	333,338	52.2	89
SI 2430/14	SC	Mixed meat sausage	Food	CFSAN107153	AAWRHO000000000.1	SRX9368263	56	4,884,337	321,380	52.3	73
SI 2461/14	SC	Chicken carcass	Food	CFSAN107154	AAWRGI000000000.1	SRX9368325	65	4,883,413	333,338	52.3	68
SI 2463/14	SC	Chicken carcass	Food	CFSAN107155	AAYKFK000000000.1	SRX9741196	68	4,914,689	263,497	52.1	77
SI 2548/14	RS	Chicken feces	Animal	CFSAN107156	AAWRDS000000000.1	SRX9368800	42	4,920,125	285,983	52.3	96
SI 3836/14	RS	Dragging swab	Environment	CFSAN107160	AAXBHC000000000.1	SRX9424059	33	4,757,086	269,483	52.5	86
SI 4882/14	MG	Chicken carcass	Food	CFSAN107164	AAXBHW000000000.1	SRX9423829	61	4,896,419	193,542	52.4	173
SI 4892/14	MG	Chicken wings	Food	CFSAN107165	AAXAKM000000000.1	SRX9423600	62	4,834,947	201,530	52.4	133
SI 4895/14	MG	Chicken carcass	Food	CFSAN107166	AAXAKH000000000.1	SRX9423603	51	4,758,951	204,497	52.3	79
SI 4901/14	MG	Pig snout	Food	CFSAN107167	AAXAKN000000000.1	SRX9423596	60	4,812,020	193,525	52.3	95
SI 5247/14	MG	Chicken upper leg and thigh	Food	CFSAN107168	AAXAKJ000000000.1	SRX9423601	67	4,785,236	158,957	52.4	88
SI 342/15	SC	Swine heart	Food	CFSAN107171	AAXHSY000000000.1	SRX9518411	83	4,877,593	263,497	52.2	111
SI 444/15	SC	Pork filet	Food	CFSAN107172	AAXHRH000000000.1	SRX9518415	78	4,841,826	221,155	52.2	97
SI 447/15	SC	Smoked and salted pork meat	Food	CFSAN107173	AAXHRI000000000.1	SRX9518412	87	4,948,504	221,155	52.2	121
SI 1809/15	SC	Meat animal meal	Animal rations	CFSAN107179	AAXHSE000000000.1	SRX9518297	70	4,854,355	201,981	52.3	118
SI 1816/15	SC	Poultry viscera animal meal	Animal rations	CFSAN107180	AAXHVG000000000.1	SRX9517669	90	4,813,298	221,155	52.3	79
SI 2280/15	SC	Chicken carcass	Food	CFSAN107182	AAXHUK000000000.1	SRX9517678	78	4,868,375	221,155	52.2	85
SI 2302/15	SC	Cleaning wipe	Environment	CFSAN107183	AAXHUC000000000.1	SRX9517686	52	4,676,437	221,155	52.3	84
SI 2370/15	SC	Carcass cleaning wipe	Food	CFSAN107185	AAXHUH000000000.1	SRX9517683	58	4,703,025	216,611	52.3	77
SI 2869/15	MG	Chicken upper leg	Food	CFSAN107190	AAXHUP000000000.1	SRX9517673	35	4,684,214	251,956	52.3	70
SI 3056/15	MG	Chicken carcass	Food	CFSAN107193	AAXHUJ000000000.1	SRX9517680	57	4,842,379	201,981	52.3	97
SI 4764/15	SC	Cleaning wipe	Environment	CFSAN107197	AAXHVH000000000.1	SRX9517653	73	4,809,343	263,613	52.1	54
SI 5391/15	SC	Disposable shoe cover	Environment	CFSAN107200	AAXHUD000000000.1	SRX9517693	52	4,816,351	263,497	52.1	79
SI 5837/15	SC	Disposable shoe cover	Environment	CFSAN107201	AAXHTN000000000.1	SRX9518269	53	4,682,394	203,759	52	72
SI 5853/15	SC	Disposable shoe cover	Environment	CFSAN107202	AAXJLL000000000.1	SRX10107873	31	4,646,034	333,177	52	36
SI 5859/15	SC	Disposable shoe cover	Environment	CFSAN107203	AAXHWB000000000.1	SRX9517633	34	4,826,566	332,890	52.3	89
SI 5911/15	SC	Cleaning wipe	Environment	CFSAN107204	AAXHVK000000000.1	SRX9517646	56	4,785,749	221,153	52.2	64
SI 5912/15	SC	Cleaning wipe	Environment	CFSAN107205	AAYKGL000000000.1	SRX9741109	60	5,039,845	274,647	52.1	161
SI 5915/15	SC	Cleaning wipe	Environment	CFSAN107206	AAYKGJ000000000.1	SRX9741110	60	5,048,568	268,897	52.2	141
SI 5923/15	SC	Cleaning wipe	Environment	CFSAN107207	AAYKGQ000000000.1	SRX9741100	58	5,084,316	332,890	52.2	185
SI 220/16	SC	Cleaning wipe	Environment	CFSAN107212	AAYKGB000000000.1	SRX9741163	91	4,808,674	191,734	51.1	83
SI 3687/16	SC	Chicken carcass	Food	CFSAN107222	AAYKGA000000000.1	SRX9741167	73	5,193,987	333,338	52.1	206
SI 4447/16	SC	Pork sausage	Food	CFSAN107224	AAYKGC000000000.1	SRX9741136	60	4,741,274	263,530	51.9	44
SI 5946/16	SC	Pork rib	Food	CFSAN107226	AAYAAA000000000.1	SRX9706883	71	4,781,146	263,497	52.1	89
SI 6987/16	MA	Human feces	Human	CFSAN107229	AAYAIC000000000.1	SRX9707272	49	4,849,107	221,494	52.4	99
SI 7876/16	RS	Human feces	Human	CFSAN107233	AAYAFO000000000.1	SRX9706835	94	4,961,253	193,312	52	109
SI 11/17	PR	Dragging swab	Environment	CFSAN107235	AAYARD000000000.1	SRX9707250	51	4,712,369	203,759	52.4	127
SI 23/17	PR	Dragging swab	Environment	CFSAN107237	AAYAFK000000000.1	SRX9706836	52	4,696,020	201,530	52.4	94
SI 238/17	PR	Dragging swab	Environment	CFSAN107238	AAYAFN000000000.1	SRX9706831	48	4,762,239	226,754	52.3	96
SI 872/17	MG	Chicken carcass	Food	CFSAN107239	AAYAFR000000000.1	SRX9706827	37	4,641,528	396,623	52.1	61
SI 1171/17	SP	Soil	Environment	CFSAN107242	AAYAFL000000000.1	SRX9706832	52	4,596,651	183,794	52.2	49
SI 1256/17	SP	Soil	Environment	CFSAN107243	AAYAFP000000000.1	SRX9706830	54	4,580,506	186,879	52.2	39
SI 2580/17	SC	Human feces	Human	CFSAN107259	AAYKFO000000000.1	SRX9741190	72	4,866,248	203,746	52.2	140
SI 2953/17	GO	Human fecal swab	Human	CFSAN107261	AAYKFZ000000000.1	SRX9741178	39	4,843,782	263,497	52.4	126
SI 2954/17	GO	Human fecal swab	Human	CFSAN107262	AAYKFE000000000.1	SRX9741199	38	4,916,543	397,894	52.4	223
SI 3380/17	GO	Human fecal swab	Human	CFSAN107263	AAYKFP000000000.1	SRX9741187	51	4,691,340	203,759	52.3	112
SI 3877/17	MG	Chicken wings	Food	CFSAN107264	AAYKFX000000000.1	SRX9741181	38	4,958,292	263,497	52.3	205
SI 3906/17	SP	Sieve residue	Environment	CFSAN107265	AAYKFS000000000.1	SRX9741183	59	4,695,925	221,498	52.3	57
SI 4065/17	PR	Human feces	Human	CFSAN107266	AAYKFR000000000.1	SRX9741189	60	4,845,749	333,338	52.2	102
SI 4067/17	PR	Human feces	Human	CFSAN107267	AAYKGD000000000.1	SRX9741162	84	5,080,992	263,497	52.3	296
SI 4069/17	PR	Human blood	Human	CFSAN107268	AAYKFD000000000.1	SRX9741200	39	4,692,193	397,347	52.3	95
SI 52/18	MG	Chicken carcass	Food	CFSAN107270	AAYKFI000000000.1	SRX9741194	48	4,767,821	285,983	52.2	96
SI 331/18	GO	Human fecal swab	Human	CFSAN107273	AAYKFT000000000.1	SRX9741182	57	4,586,964	204,497	52.4	86
SI 623/18	SC	Human feces	Human	CFSAN107279	AAYKFY000000000.1	SRX9741179	82	4,604,903	141,972	52.2	52
SI 661/18	MS	Human feces	Human	CFSAN107280	AAYKFW000000000.1	SRX9741180	52	4,596,946	194,623	52.2	47
SI 942/18	RS	Human fecal swab	Human	CFSAN107281	AAYKFM000000000.1	SRX9741192	68	4,981,050	263,497	52.3	128
SI 1634/18	SC	Yellowtail amberjack fish meat	Food	CFSAN107284	AAYKFQ000000000.1	SRX9741185	35	4,641,031	397,894	52.3	84
SI 2676/18	GO	Avian reproductive matrix	Animal	CFSAN107285	AAYKFF000000000.1	SRX9741197	56	4,803,122	240,256	52	84

a RS, Rio Grande do Sul; PR, Paraná; SC, Santa Catarina; SP, São Paulo; MG, Minas Gerais; MS, Mato Grosso do Sul; GO, Goiás; BA, Bahia; AL, Alagoas; PE, Pernambuco; MA, Maranhão.

b Cleaning wipe: a material similar to the synthetic cloths sold commercially for domestic cleaning; used in the microorganism isolation procedure on industry and farm facility surfaces in Brazil.

## References

[1] Majowicz SE, Musto J, Scallan E, Angulo FJ, Kirk M, O’Brien SJ, Jones TF, Fazil A, Hoekstra RM, International Collaboration on Enteric Disease “Burden of Illness” Studies. 2010. The global burden of nontyphoidal *Salmonella* gastroenteritis. Clin Infect Dis 50:882–889. doi:10.1086/650733. 20158401

[2] Shahada F, Sugiyama H, Chuma T, Sueyoshi M, Okamoto K. 2010. Genetic analysis of multi-drug resistance and the clonal dissemination of beta-lactam resistance in *Salmonella* Infantis isolated from broilers. Vet Microbiol 140:136–141. doi:10.1016/j.vetmic.2009.07.007. 19665854

[3] Almeida F, Pitondo-Silva A, Oliveira MA, Falcão JP. 2013. Molecular epidemiology and virulence markers of *Salmonella* Infantis isolated over 25 years in São Paulo State, Brazil. Infect Genet Evol 19:145–151. doi:10.1016/j.meegid.2013.07.004. 23860124

[4] Brown AC, Chen JC, Watkins LKF, Campbell D, Folster JP, Tate H, Wasilenko J, Van Tubbergen C, Friedman CR. 2018. CTX-M-65 extended-spectrum β-lactamase-producing *Salmonella enterica* serotype Infantis, United States. Emerg Infect Dis 24:2284–2291. doi:10.3201/eid2412.180500. 30457533 PMC6256390

[5] Ranjbar R, Rahmati H, Shokoohizadeh L. 2018. Detection of common clones of *Salmonella enterica* serotype Infantis from human sources in Tehran hospitals. Gastroenterol Hepatol Bed Bench 11:54–59. doi:10.22037/ghfbb.v0i0.1202. 29564066 PMC5849119

[6] Campioni F, Falcão JP. 2013. Genotypic diversity and virulence markers of *Yersinia enterocolitica* biotype 1A strains isolated from clinical and non-clinical origins. APMIS 122:215–222. doi:10.1111/apm.12126. 23763723

[7] Timme RE, Wolfgang WJ, Balkey M, Venkata SLG, Randolph R, Allard M, Strain E. 2020. Optimizing open data to support One Health: best practices to ensure interoperability of genomic data from bacterial pathogens. One Health Outlook 2:20. doi:10.1186/s42522-020-00026-3. 33103064 PMC7568946

[8] Souvorov A, Agarwala R, Lipman DJ. 2018. SKESA: strategic k-mer extension for scrupulous assemblies. Genome Biol 19:153. doi:10.1186/s13059-018-1540-z. 30286803 PMC6172800

[9] Klimke W, Agarwala R, Badretdin A, Chetvernin S, Ciufo S, Fedorov B, Kiryutin B, O’Neill K, Resch W, Resenchuk S, Schafer S, Tolstoy I, Tatusova T. 2009. The National Center for Biotechnology Information’s Protein Clusters Database. Nucleic Acids Res 37:D216–D223. doi:10.1093/nar/gkn734. 18940865 PMC2686591

